# A case report of non-lamin A/C dilated cardiomyopathy presenting in a patient with Najjar–Malouf syndrome

**DOI:** 10.1093/ehjcr/ytaf110

**Published:** 2025-02-26

**Authors:** Gian Flury, Flurina Arquint

**Affiliations:** Department of Internal Medicine, Ospidal, Center da Sandà Engiadina bassa, Via da’l Ospidal, Scuol CH-7550, Switzerland; Clinic of Cardiology, Department of Internal Medicine, Kantonsspital Graubünden, Loestrasse 170, Chur CH-7000, Switzerland

**Keywords:** Heart failure with reduced ejection fraction, Syndromic cardiomyopathy, Najjar–Malouf syndrome, Cardiomyopathy, Case report

## Abstract

**Background:**

Phenotyping and genotyping of cardiomyopathies are becoming increasingly important. We describe a rare form of syndromic dilated cardiomyopathy, years after the initial diagnosis of intellectual disability and hypergonadotropic hypogonadism, known as Najjar–Malouf syndrome or cardiogenital syndrome.

**Case summary:**

A 45-year-old woman was referred for inpatient treatment because of progressive dyspnoea, chest tightness, and ankle oedema. She had a diagnosis of undefined psychomotor development delay and hypergonadotropic hypogonadism, for which she has been receiving hormone substitution treatment since she was 19 years old. The physical examination revealed signs of congestion as well as a right convex thoracic scoliosis and hyperkyphosis. Cardiac biomarkers were elevated, and echocardiography showed severe dilated cardiomyopathy with impaired systolic function, severe pulmonary hypertension, and secondary mitral regurgitation. Cardiac magnetic resonance imaging showed a severely dilated left ventricle with severely reduced left ventricular ejection fraction (20%), thinned myocardium, and late-gadolinium enhancement predominantly in the septum. Genetic screening for dilatative cardiomyopathy-associated genes revealed no mutations, in particular, no mutation of the lamin A/C (LMNA) gene. The patient progressed to heart failure with severely reduced ejection fraction 26 years after diagnosis of psychomotor development delay and hypergonadotropic hypogonadism.

**Discussion:**

The triad of intellectual disability, hypergonadotropic hypogonadism, and cardiomyopathy enabled the diagnosis of Najjar–Malouf syndrome, also known as cardiogenital syndrome. This case underscores the diagnostic and therapeutic challenges of Najjar–Malouf syndrome, emphasizing the significance of thorough evaluation and genetic testing, particularly considering the association with LMNA mutations. Further research is needed to improve understanding and management strategies for this rare syndrome.

**Learning points**

- Najjar–Malouf syndrome is a rare congenital disorder associated with dilated cardiomyopathy, intellectual disability, hypergonadotropic hypogonadism, and skeletal characteristics.
- Genetic analyses including mutations in the lamin A/C (LMNA) gene should be performed, as LMNA-associated cardiomyopathies have been described in this syndrome and have an unfavourable prognosis.

## Introduction

Cardiomyopathies involve various potential aetiologies, making the precise identification of individual phenotypes and genotypes increasingly important. Treatment decisions often depend on the specific cardiomyopathy subtype. Precision medicine, which integrates genetic, environmental, and lifestyle factors, is refining therapy selection. Moreover, advancements in molecular biology, particularly genome modification techniques, are facilitating the development of targeted treatments for specific molecular subtypes. This case report discusses a very rare form of dilated cardiomyopathy occurring in Najjar–Malouf syndrome, characterized by intellectual disability and hypergonadotropic hypogonadism. The literature search related to this unusual clinical triad led to the diagnosis of Najjar–Malouf syndrome. In the Orphanet database (Orphanet kosekschweiz.ch) for rare diseases, the term ‘cardiogenital syndrome’ is also given as a synonym^1^ (Orpha 2239). Its prevalence is estimated at <1/1 000 000.

## Summary figure

**Table:** 

Date	Age	Clinical events
1976		Spontaneous delivery at 39 weeks gestation; ‘Acidotic postnatal cardiomegaly’
1976	5 Days	Cardiac catheterization: Ventricular septal defect with 40% left-to-right shunt, no further information available
1989	13 Years	Diagnosis of a psychomotor developmental delay
1995	19 Years	Diagnosis: Hypergonadotropic hypogonadism Start of oestrogen and progestin substitution
2007	31 Years	Echocardiography: LVEF 59%, no relevant pathology
4/2022	46 Years	Echocardiography: Left ventricular ejection fraction (LVEF) 25%, eccentric left ventricular hypertrophy, left ventricular end-diastolic diameter (LVEDD) 6.3 cm; Left ventricular mass index (LVMI) 102 g/m^2^. Peak systolic right ventricular (RV) - right atrial (RA) gradient 50 mmHg; secondary mitral regurgitation; Dyspnoea NYHA IV (orthopnea). Diagnosis: Najjar–Malouf syndrome
4–11/2022		Optimal heart failure drug therapy => NYHA II
01/2023	47 Years	Cardiac magnetic resonance imaging (MRI): LVEF 20%; left ventricular indexed end-diastolic volume (LVEDVI) 175 mL/m^2^; septal dyssynchrony with left bundle branch block
03/2023		Genetic analysis: no evidence of a DCM-associated gene variant, in particular, no lamin A/C (LMNA) mutation
05/2023		Primary prophylactic implantation of 1-chamber implantable cardioverter defibrillator (ICD) [implantation of cardiac resynchronization defibrillator (CRT-D) system fails]
08/2023		ICD control: singular short non-sustained ventricular tachycardia (NSVT) episodes; exertional dyspnoea NYHA II

## Case presentation

A 45-year-old female patient was admitted with decompensated heart failure. Her medical history included postnatal cardiomegaly and a ventricular septal defect with 40% left-to-right shunt. No further information on that is available. At ages 13 and 19, she was diagnosed with psychomotor delay and hypergonadotropic hypogonadism, respectively. Substitution with oestradiol and norethisterone is then started. A first transthoracic echocardiogram at 31 was normal, despite symptoms such as rapid physical fatigue, myalgias, and arthralgias and a positive Sokolow index in the ECG. No cardiac or other relevant diseases are known in the first-degree relatives. There is no blood relationship between the parents. Original medical records for the first 19 years of her life are not available. Information about this is based on later entries in the only fragmentary medical records of the patient’s former family doctor.

The current history taking is very difficult due to the patient’s mnestic deficits. She reports that she has been suffering from thoracic tightness and dyspnoea on exertion for ‘some time’. In the last 2 weeks or so, rapid fatigability, orthopnea, ankle and eyelid oedema, and a persistent unproductive cough have also developed.

**Figure 1:**
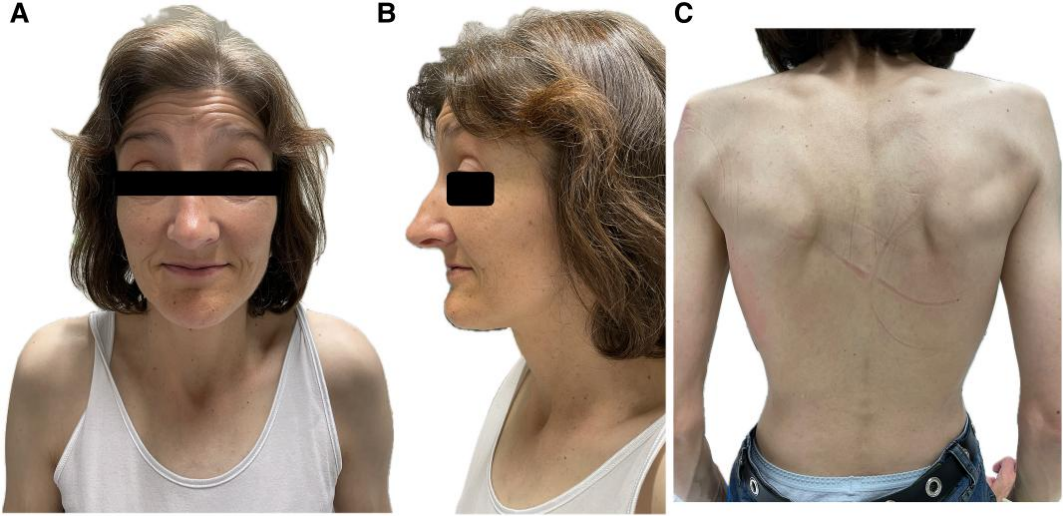
Patient with slight blepharoptosis (*A*, *B*), and right convex thoracic scoliosis and hyperkyphosis (*C*).

**Figure 2:**
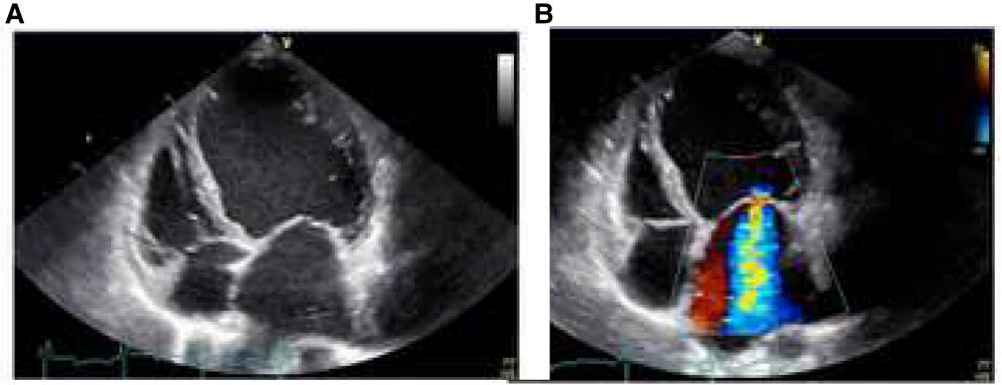
Echocardiogram. Four chamber view with severely dilated left ventricle (*A*) and moderate to severe mitral regurgitation (*B*).

**Figure 3:**
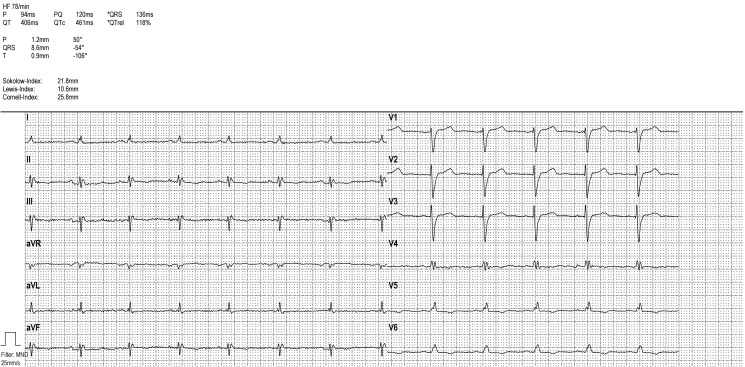
Electrocardiogram with sinusrhythm and left bundle branch block.

The physical examination revealed a blood pressure of 106/79 mmHg, a pulse of 90/min with regular basic rhythm and frequent extrasystoles. Regular heart sounds; 2/6-loud band-shaped systolic murmur with p.m. over the apex of the heart, radiating into the axilla. In addition, there were signs of cardiac decompensation with congested neck veins, pronounced ankle oedema, cool acres, and weakened breath sounds basally on both sides but no crackles. Respiratory rate was increased (24/min) and oxygen saturation was decreased (SpO2 86% on room air). The weight was 49.3 kg, height 167 cm, and BMI 17.6 kg/m^2^. In addition, there was a slight blepharoptosis, a right convex thoracic scoliosis, and hyperkyphosis (*Figure 1*). No glandular tissue was palpable on bilateral breast palpation. Axillary and pubic hair was sparse. The patient scored 22/30 points in the Montreal Cognitive Assessment (MoCa) test and showed difficulties mainly in language and memory (MoCa, normal ≥26 points). The only conspicuous laboratory findings were an elevated high-sensitivity troponin I (57.4 ng/L; *N* < 19) and N-terminal pro-B-natriuretic peptide (5406 pg/mL; *N* < 450). Transthoracic echocardiography showed a dilated left ventricle (LVEDD 6.3 cm, LVMI 102 g/m^2^; relative wall thickness 0.16) with severely impaired systolic function (LVEF biplan according to Simpson 25%; Global Longitudinal Strain −7%), a normal-sized right ventricle with normal systolic function and an enlarged left atrium (LAVI 41 mL/m^2^). Furthermore, there was moderate to severe secondary mitral regurgitation and severe pulmonary hypertension: RV–RA 50 mmHg; estimated right atrial pressure of ∼15 mmHg (*Figure 2*, see Supplementary material online, *Videos S1* and *S2*). The electrocardiogram showed a complete left bundle branch block (LBBB) with signs of low voltage (*Figure 3*). The amplitude criteria for left ventricular hypertrophy described electrocardiographically in the 31-year-old patient were no longer present in the current ECG. We assume that the ECG morphology, in particular, the now complete LBBB, has changed in parallel with the dilated cardiomyopathy that has developed in the meantime. Transvaginal ultrasound was inconclusive in virgo intacta (penetration possible only to the hymen). The transabdominal ultrasound showed an antivertic uterus, both ovaries could not be visualized. Overall, the following special constellation was found in the patient:

- Severe dilated cardiomyopathy (unknown aetiology)
- Hypergonadotropic hypogonadism (currently under hormone substitution)
- Intellectual disability

This triad led to the diagnosis of Najjar–Malouf syndrome, also known as cardiogenital syndrome.

Guidelines-compliant drug therapy for heart failure was established, with bisoprolol, sacubitril/valsartan, spironolactone, empaglifozin, and torasemide with gradual up-titration. Outpatient cardiological follow-up 2 and 7 months after hospital discharge showed a clinically cardiac-compensated patient on optimal heart failure medication. She reported significantly better performance in everyday life and continued to have NYHA II exertional dyspnoea. The follow-up echocardiograms 2 and 7 months later showed a successive decrease in LVEDD (from 6.3 to 5.9 cm), in systolic pulmonary pressure (from 65 to 38 mmHg) and in mitral regurgitation (now mild), but an unchanged severely impaired left ventricular ejection fraction. Cardiac MRI 9 months later revealed a severely dilated left ventricle (LVEDVI 175 mL/m^2^) with severely reduced LVEF (20%) and generalized hypokinesia as well as septal dyssynchrony with complete LBBB.

**Figure 4:**
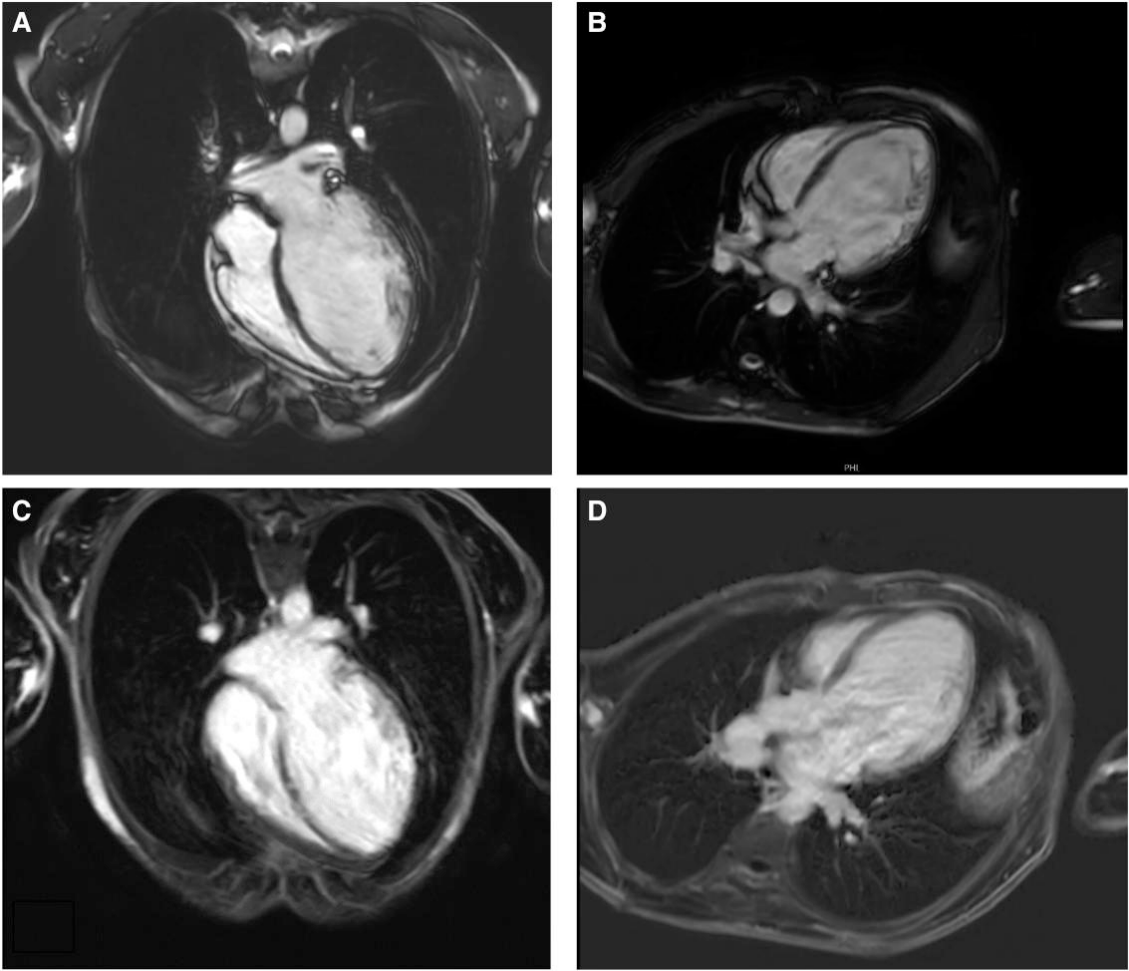
Cardiac magnetic resonance: cine steady-state free-procession (SSFP) images of the four chamber (*A*) and three chamber view (*B*). Late-gadolinium enhancement images show midwall enhancement, primarily in the mid-septum and mid-apical inferobasal wall (*C* + *D*).

**Figure 5:**
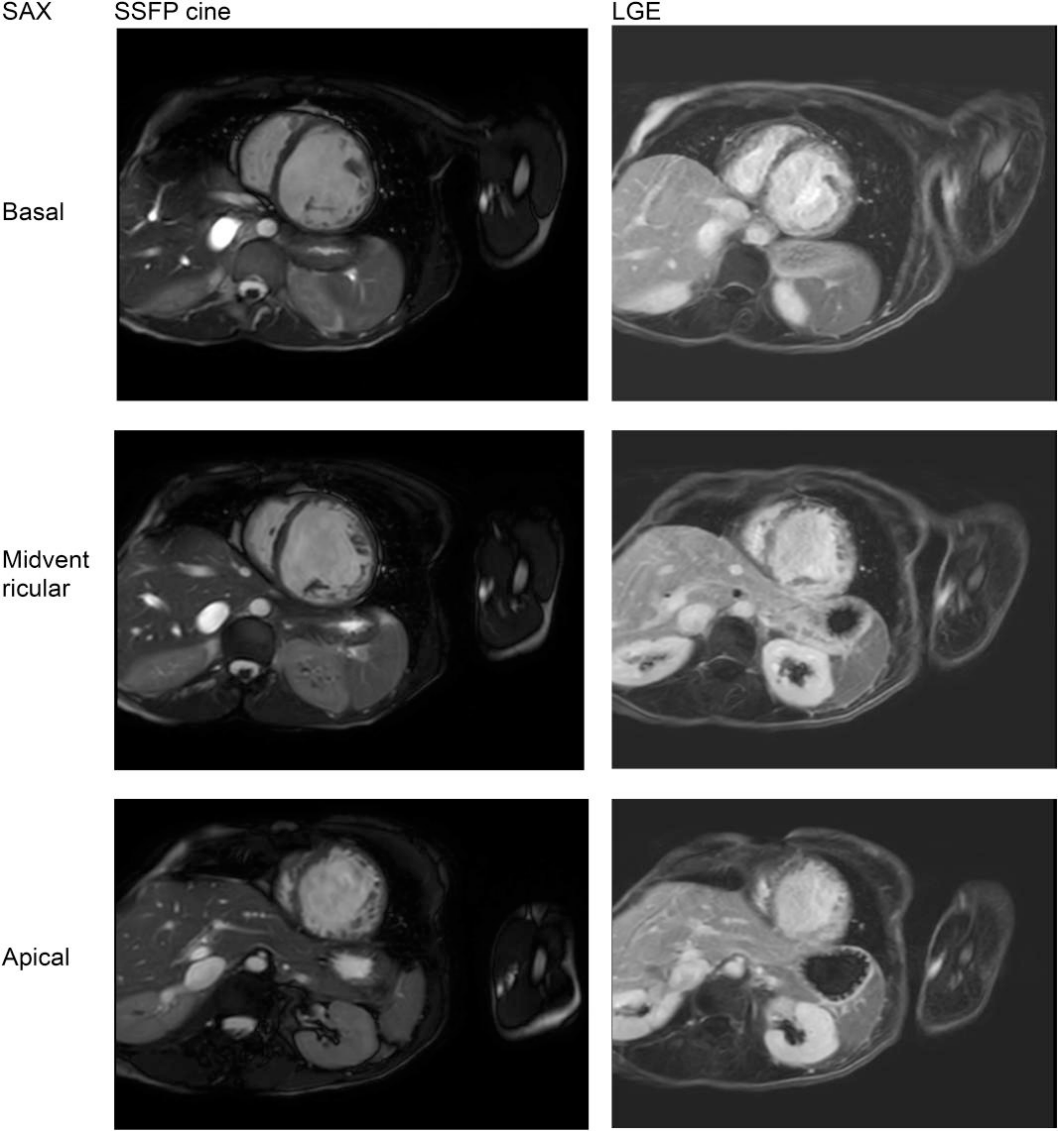
Cardiac magnetic resonance: cine steady-state free-procession (SSFP) images of the short axis view (left panel). Late-gadolinium enhancement images show midwall enhancement, primarily in the mid-septum and mid-apical inferobasal wall (right panel).

Furthermore, the myocardium of the left ventricle was severely thinned with irregular endocardial surface and excessive trabecularization, with midwall late-gadolinium enhancement, primarily in the mid-septum and mid-apical inferobasal wall (*Figure 4*, see also Supplementary material online, *Figure 5* and Supplementary material online, *Videos S3–S6*). The indexed myocardial left ventricular mass on MRI was slightly increased (69 g/m^2^, normal 30–68). There was also a prolongation of the native myocardial T1 relaxation time.

Genetic screening for dilatative cardiomyopathy (DCM)-associated genes revealed no mutations, in particular no mutation of the lamin A/C (LMNA) gene. In total, the following genes were tested: ABCC9, ACTA2, ACTC1, ACTN2, ANKRD1, BAG3, CSRP3, DES, DMD, DSC2, DSG2, DSP, EMD, FLNC, GATAD1, HFE, ILK, JUP, JPH2, LAMP2, LMNA, LDB3, MYBPC3, MYH6, MYH7, MYL2, MYPN, MYL3, NEXN, NKX2-5, PKP2, PLN, PRDM16, PRKAG2, RBM20, SGCD, TCAP, TJP1, TMEM43, TNNC1, TNNI3, TNNT2, TPM1, TTN, TTR, SCN5A, VCL.

In the absence of (i) improvement in left ventricular function under optimal heart failure therapy and (ii) ventricular dyssynchrony with an increase in QRS duration to 138 ms due to a LBBB, the decision was made to implant a cardiac resynchronization defibrillator (CRT-D system) after detailed discussion with the patient and her two siblings. However, placement of the left ventricular lead was not successful for anatomical and technical reasons, which is why only a right ventricular single-chamber ICD was implanted for primary prophylaxis. Since then, there has been a stable clinical course with persistent exertional dyspnoea NYHA II with single short non-sustained ventricular tachycardias in the ICD memory.

The patient currently lives alone, cares for herself completely independently, and is only assisted by her two siblings with more complex administrative tasks (e.g. tax returns, disability pension applications, exceptional banking matters, etc.). According to the patient herself, her relatives, and her family doctor, there has been no change in her cognitive abilities since she became an adult. Based on the results of the MoCa test, there is only mild cognitive impairment (22/30 points; 18–25 points = mild impairment). There is no specific treatment for cognitive impairment. With regard to the management of heart failure, these mild cognitive deficits have so far only made communication more difficult, which is significantly more time-consuming (e.g. explaining the understanding of cardiac pathology, the need for polymedication, and the indication for ICD implantation). Compliance with medication has been excellent so far, but requires constant re-explanation of why it must be continued indefinitely. Since hormone replacement, the patient has been free of symptoms of hypergonadotropic hypogonadism. She is sexually inactive (virgo intacta). There is no discernible effect on the cardiomyopathy.

The patient measures and records her body weight daily. Clinical and laboratory tests (including NT-proBNP, electrolytes, renal function) are carried out by the general practitioner every 6–8 weeks, or earlier if there are signs of deterioration in cardiac function (shortness of breath, increasing exercise intolerance, peripheral oedema, rapid weight gain). With a stable course to date, the ICD is currently checked every 6 months and echocardiograms are performed annually. The last one was done after submission of the manuscript and showed further stable results (LVEF 28%; GLS-12%). Spiroergometry to objectify exercise capacity (VO2max) is planned for the next cardiological follow-up. If there is clinical, laboratory, or echocardiographic evidence of the progression of heart failure, another attempt to implant a CRT or conduction system pacing will be discussed with the patient, especially if the LBBB is increasing in duration. Finally, heart transplantation should be considered in the medium term for this relatively young patient.

## Discussion

We present a sporadic case of a young female patient with dilated cardiomyopathy, hypergonadotropic hypogonadism, and intellectual disability. S.S. Najjar first described a syndromic triad with incompletely developed external genitalia, intellectual disability, and undefined cardiomyopathy in two Lebanese brothers in 1973.^2^ This was followed in 1984 by another publication by the same authors describing three other brothers with the same triad.^3^ In 1985, J. Malouf published the case reports of two sisters with secondary hypergonadotropic hypogonadism and ovarian dysgenesis as well as ‘congestive cardiomyopathy’ and postulated an autosomal recessive inheritance based on the familial pedigree.^4^ In each of these case reports, the parents were related by blood. In a literature review, we found 14 case reports to date with a total of 22 patients with similar phenotypes. The majority of these are case reports with one to three cases, plus a summarized overview of 16 of the published cases.^3^

Information on echocardiographic findings is only available in publications after 1984. As in our patient, most cases showed eccentric left ventricular hypertrophy with moderate to severe diffuse LVEF impairment. In most patients, the right ventricle was morphologically unremarkable and normally contractile; isolated cases of right ventricular dysfunction have been described. Some cases also showed left atrial enlargement and functional mitral regurgitation. In most published cases, heart failure manifested in adolescence or early adulthood and cardiomyopathy was diagnosed.^5–7^ In contrast, in our patient, echocardiography at the age of 31 years showed normal findings, and a cardiomyopathy was manifested and diagnosed at the age of 46 years. General statements about the long-term prognosis of patients with Najjar–Malouf syndrome cannot be made reliably because of the very small number of cases worldwide, the lack of publications on the long-term course, and possibly also because of the considerable increase in the armamentarium for the treatment of heart failure and arrhythmias in recent years (drug therapy, ICD, CRT).

Najjar–Malouf syndrome affects women and men and, despite a similar clinical phenotype, has no uniform genotype. Facial dysmorphisms (e.g. narrow face and chin, blepharoptosis, broad nasal root), skin and bone changes (e.g. kyphoscoliosis), and occasionally a marfanoid habitus and lipodystrophy are also frequently associated. Some cases are associated with mutations of the LMNA,^6^ in others, such as ours, no LMNA mutation can be detected.^7^ Lamin A/C mutations cause laminopathies, a group of diseases with different phenotypes, including certain muscular dystrophies, neuropathies, premature aging, lipodystrophies, and cardiomyopathies.^8^ Lamin A/C-associated cardiomyopathies have an unfavourable prognosis with early onset of conduction disturbances, malignant arrhythmias, and usually rapidly progressive heart failure.^9^ The 2023 ESC guidelines for the management of cardiomyopathies recommend genetic testing in apparently sporadic dilated cardiomyopathy for the direct benefit of the patient: to confirm the diagnosis, where it may inform prognosis, where it may inform treatment selection, or where it may inform their reproductive management.^9^ If a pathogenic LMNA mutation is detected, ICD implantation should be based on the LMNA 5-year risk score and the presence of additional factors such as LVEF <50%, NSVT, or atrioventricular conduction delay.

This case report shows that when cardiomyopathy occurs in combination with diseases of other organ systems, a syndromic aetiology should be considered, whereby the cardiomyopathy—as in our patient—can unmask itself many years after other clinical manifestations.

Najjar and Malouf first described cases with suspected autosomal recessive inheritance based on several sibships (cousins) with unaffected parents, but the total number of patients is small and no genetic analysis was performed. Subsequent isolated cases suggested new mutations; genetic analysis revealed variants, including in the LMNA gene, associated with different phenotypes, suggesting diverse genotype–phenotype relationships in Najjar–Malouf syndrome.^6^ Genetic diagnosis, particularly for mutations in the LMNA gene, is indicated for prognostic and therapeutic reasons.

Due to the rarity of the cases and their likely high genotypic–phenotypic diversity, a promising research strategy for non-LMNA-associated cases of Najjar–Malouf syndrome is difficult to define. One possibility would be to establish an international registry for currently living and future newly diagnosed patients with this rare syndrome. This registry would need to be based in a university research centre with appropriate infrastructure and financial resources to search for specific genetic mutations associated with cardiomyopathy-associated genes, similar to Vaikhanskaya’s studies. Such a registry would also allow long-term follow-up to assess medium- and long-term prognosis and the appropriate timing of specific therapeutic interventions (e.g. HTPLX). Data on LMNA-associated cardiomyopathies are already available and have led to diagnostic and therapeutic recommendations in the ESC guidelines.^9^

If dilated cardiomyopathy is found in a young woman, the presence of hypergonadotropic hypogonadism should be considered. This includes delayed or absent sexual maturity with incomplete or absent development of the sexual phenotype, amenorrhoea, and possibly infertility. Diagnostic evidence includes elevated gonadotropins (luteinizing hormone, follicle stimulating hormone), reduced oestrogen levels, and pathological sonographic findings of the reproductive organs.

In the differential diagnosis of Najjar–Malouf syndrome, other genetic syndromes such as the much more common Noonan syndrome or Turner syndrome should be considered. In Noonan syndrome, which is usually sporadic, there may be a combination of mild cognitive deficit with congenital heart defects and hypertrophic cardiomyopathy. In Turner syndrome, there may be a combination of congenital heart defects and ovarian dysfunction with increased gonadotropin and decreased oestrogen levels, but no cognitive impairment. In both syndromes, the phenotypic presentation (e.g. short stature, hypertelorism, other musculoskeletal abnormalities) is markedly different from that of Najjar–Malouf syndrome.

## Lead author biography


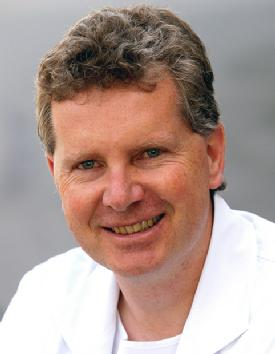


Dr Gian Flury is currently a senior consultant at the Medical Department of the Engiadina Bassa Regional Hospital. He was head of this department until 2023. His cardiology focus is on heart failure and non-invasive rhythmology.

## Supplementary Material

ytaf110_Supplementary_Data

## Data Availability

The data underlying this article will be shared on reasonable request to the corresponding author.
